# Change in the association of body mass index and systolic blood pressure in Germany – national cross-sectional surveys 1998 and 2008–2011

**DOI:** 10.1186/s12889-015-2023-8

**Published:** 2015-07-25

**Authors:** Carolin Adler, Angelika Schaffrath Rosario, Claudia Diederichs, Hannelore K. Neuhauser

**Affiliations:** Department of Epidemiology and Health Monitoring, Robert Koch Institute, General Pape Str. 62-66, Berlin, D-12101 Germany; DZHK (German Center for Cardiovascular Research), partner site, Berlin, Germany

**Keywords:** Body mass index, Systolic blood pressure, Association, Germany, Adults, Representative, Health examination survey

## Abstract

**Background:**

A recent weakening and even decoupling of the association of body mass index (BMI) and systolic blood pressure (SBP) in population data was reported, i. a. for Western Europe.

**Methods:**

The association of BMI and SBP in recent cross-sectional population data from Germany was investigated in participants aged 18–79 years with BMI 17.5-40 kg/m^2^ from national health examination surveys 1998 (*n* = 6,931) and 2008–2011 (*n* = 6,861) in Germany. The association was analyzed both in the overall samples and in participants without antihypertensive medication.

**Results:**

From 1998 to 2008–11, age- and sex-standardized mean SBP decreased from 129.0 (CI 128.2-129.7) to 124.1 (123.5-124.6) mmHg in all participants and from 126.0 (125.4-126.7) to 122.3 (121.7-122.8) mmHg among persons not on antihypertensive medication. The proportion of persons treated with antihypertensives augmented from 19.2 % (17.7-20.8) to 25.3 % (24.0-26.6). Mean BMI remained constant at around 27 kg/m^2^ with a slight increase in obesity prevalence. BMI was positively associated with SBP both in 1998 and 2008–11, yet the association tended to level out with increasing BMI suggesting a non-linear association. The strength of the BMI-SBP-association decreased over time in all and untreated men. In women, the association weakened in the overall sample, but remained similarly strong in untreated women. The unadjusted linear regression models were used to estimate the increase in SBP within 5-unit BMI increases. E. g. for men in 1998, SBP was higher by 7.0 mmHg for a BMI increase from 20 to 25 kg/m^2^ and by 3.6 mmHg for BMI 30 to 35 kg/m^2^. The corresponding values for 2008–11 were 3.8 mmHg and 1.7 mmHg.

**Conclusions:**

The cross-sectional association of BMI and SBP decreased between 1998 and 2008–11 in Germany, however it did not disappear and it is in part explained by improvements in the diagnosis and treatment of high blood pressure.

**Electronic supplementary material:**

The online version of this article (doi:10.1186/s12889-015-2023-8) contains supplementary material, which is available to authorized users.

## Background

High blood pressure (BP) represents the leading single risk factor for overall mortality and burden of disease globally [1, 2]. In 2001, 17.6 % of all premature deaths as well as 9.3 % of disability-adjusted-life-years (DALYs) were attributable to raised BP [3] and more than one third of the population in high-income countries had hypertension [2].

A positive association of overweight and BP has been reported from numerous cross-sectional and prospective studies over many decades [4, 5]. The INTERSALT study, which included cross-sectional data from 32 countries, estimated that after multivariable adjustment a one-unit increase in body mass index (BMI) was associated with a 0.91 mmHg systolic blood pressure (SBP) increase in men and 0.72 mmHg in women [6].

During the last decades, opposite population trends in the development of BP and BMI have been observed in many countries. While the prevalence of overweight and obesity was still rising or remained constant on a high level, i.e. in the U.S. [7] and most Western European countries [8] including Germany [9], BP declined in these populations during the same period [10–12]. This suggests that the strength of the BMI-SBP-association may have decreased over time. Most recently, even a dissociation of BP from BMI at the population level was reported: the Global Burden of Metabolic Risk Factors of Chronic Diseases Collaborating Group, with country-level risk estimates from 199 countries, showed a positive cross-sectional association of SBP and BMI for western European men and women in 1980, but no association with 2008 data [13]. This study could not account for antihypertensive medication use which increased worldwide in these decades, thus truncating the BP distribution and diminishing the SBP-BMI-correlation, particularly in the obese.

However, a significantly decreasing BMI-SBP-association was also reported for persons not on antihypertensive treatment from two cross-sectional surveys conducted in 1989 and 2004 at the Seychelles [14]. Similarly, data from seven population-based studies with 9- to 11-year old U.K. children found a weakening in the association, too [15]. Additional indirect evidence was given by an analysis of data from the National Health and Nutrition Examination Survey (NHANES) conducted among adults and children of the United States, where the prevalence of high BP decreased more in overweight and obese persons than in lean persons over time, although a survey x BMI group interaction was not significant indicating that the declines in high BP did not differ by BMI [16].

The aim of the present study was to investigate changes in the cross-sectional BMI-SBP-association in the general population in Germany between 1998 and 2008–2011 taking into account the impact of an increased use of antihypertensive medication. Therefore, data from two population-based national health surveys, the *German National Health Interview and Examination Survey 1998 (GNHIES98)* and the *German Health Interview and Examination Survey for Adults 2008–11 (DEGS1)* were analysed, both the overall samples and the subsamples of participants not taking antihypertensive medication.

## Methods

### Study population

Two national health examination surveys were conducted in Germany in 1998 and 2008–2011. Both used a nationwide two-stage clustered sample design with selection of study points based on community type and federal state and subsequent sampling of persons aged 18–79 years stratified by sex and age group from the local population registers [17, 18]. The German Health Interview and Examination Survey for Adults (DEGS1) 2008–2011 comprised a total of *n* = 7,115 persons examined at one of 180 study points, of whom *n* = 4,192 were first-time participants (response: 42 %) and *n* = 2,923 (response: 62 %) were former participants of the German National Health Interview and Examination Survey 1998 (GNHIES98). The net sample of the GNHIES98 consisted of *n* = 7,124 persons (response: 61 %) from 120 study points. The study was approved by the ethical committee of Charité University Medicine, Berlin, and by the Federal Commissioner for Data Protection and Freedom of Information. Informed written consent and assent were obtained from all participants.

### Measurement and survey methods

BP was measured according to a standardized protocol which was almost identical in both surveys, except that the standard mercury sphygmomanometer (Erkameter 3000, Bad Tölz, Germany) employed in the GNHIES98 was replaced by an automated oscillometric Datascope Accutorr Plus device (Datascope Accutorr Plus, Mahwah, NJ, USA) together with a new set of manufacturer-provided cuffs and adapted cuff-selection-rules. The participants sat quiet and upright on a height-adjustable chair with their back supported, the right forearm was resting on a table at heart level, elbow slightly bent, legs uncrossed and feet firmly on the floor. The correct cuff size was determined with the upper arm circumference (AC) measured half way between the acromion and the olecranon. Both surveys used three cuff sizes. The cuff bladder dimensions (width × length) in DEGS1 were: 10.5 × 23.9 cm for ACs of 21–27.9 cm, 13.5 × 30.7 cm for ACs of 28–35.9 cm and 17 × 38.6 cm for ACs of 36–46 cm. The corresponding sizes in GNHIES98 were: 8 × 20 cm for ACs < 20 cm, 12 × 28 cm for ACs of 20–40 cm and 14 × 40 cm for ACs > 40 cm. The correct position of the cuff above the brachial artery was ensured with a mark on the cuff. Three blood pressure measurements were taken at 3-min intervals, following an initial 5-min resting period (GNHIES98: 3 min) after a non-strenuous part of the examination.

The participants were asked to bring along their medication of the previous 7 days and antihypertensive medication use was defined according to the WHO Anatomic Therapeutic Chemical Classification System (ATC): antihypertensive drugs (C02), diuretics (C03), beta-blockers (C07), calcium channel blockers (C08) and ACE inhibitors (C09). Hypertension was defined as: SBP ≥140 mmHg or DBP ≥90 mmHg or treatment with ATC-coded antihypertensive medication. However, the antihypertensive medication was only used for defining hypertension if the participants reported having hypertension since the indication for taking these drugs may be other than hypertension.

Body height and weight measurements also followed standardized procedures with the participants dressed only in underwear without shoes. Body height was measured with a portable stadiometer (Holtain Ltd., UK, precision: 0.1 cm) and body weight with a calibrated electronic scale (SECA, column scale 930, precision: 0.1 kg). BMI was calculated as weight in kilogram (kg) divided by height in meter squared (m^2^) and BMI was used to define non-overweight (BMI < 25 kg/m^2^), overweight (BMI ≥ 25 to <30 kg/m^2^) and obesity (BMI ≥ 30 kg/m^2^).

Information on lifestyle and socio-demographic variables were obtained with a self-administered questionnaire. Social status was determined using an index with information on school education and vocational training, occupational status and net household income (weighted by household needs) permitting classification into low, middle and high status groups [19]. Alcohol consumption was calculated in gram/day (g/d) on basis of questions about consumption frequency and amount of beer, light beer, alcohol-free beer, wine and liquor. Alcohol in g/day was then divided into three classes according to German guidelines on tolerable upper intake levels: non-drinker (0 g/d), light drinker (men: >0 to 20 g/d, women: >0 to 10 g/d) and heavy drinker (men: >20 g/d, women: >10 g/d) [20]. Smoking status was assessed by smoking frequency (daily, occasionally, no longer, never) and amount of cigarettes smoked per day and was categorized into current daily smoker (≥1 cigarette per day) or non-smoker (including occasional smoker and ex-smoker). Sports activity was asked with “How often do you exercise?” and the response items were: “no sports activity”, “<1 h/week”, “1-2 h/ week”, “2-4 h/week” and “>4 h/week”. This information was subsumed into three groups: no sports activity, sports activity <2 h/week and sports activity >2 h/week.

### Analysis

Analyses were performed in 18–79 year old GNHIES98 and DEGS1 participants. Exclusion criteria were missing information on BP (GNHIES98: 0.2 %, DEGS1: 0.3 %), antihypertensive medication use (GNHIES98: 0.4 %, DEGS1: 0.3 %) or BMI (GNHIES98: 0.7 %, DEGS1: 0.7 %). In addition, BMI outliers <17.5 kg/m^2^ or >40 kg/m^2^ (GNHIES98: *n* = 193, 2.7 %; DEGS1: *n* = 215, 3 %) were excluded since the aim of the study was to describe the BP-BMI association over a BMI range that is common in the general population, while at the lowest and highest extremes of BMI a ceiling effect on BP is likely. Analyses were first run for all participants (*n* = 6,931 for GNHIES98 and *n* = 6,861 for DEGS1), then for participants without antihypertensive medication as defined above (*n* = 5,663 for GNHIES98 and 4,755 for DEGS1).

The average of the second and third blood pressure measurements were used for analysis. The GNHIES98 BP data were calibrated for comparison with DEGS1 data based on a formula from a methodological study described previously [21]. In brief, the GNHIES98 and DEGS1 BP protocols were compared according to the principles of the International Protocol revision 2010 for the validation of blood pressure measuring devices in adults of the European Society of Hypertension in a measurement sequence with 105 participants yielding 315 measurement pairs. SBP and DBP values were higher with the mercury sphyghmomanometer in the GNHIES98 protocol as compared to the Datascope measurements with the DEGS1 protocol. Measurement differences increased with BP, pulse pressure, the difference in the ratio of cuff width to arm circumference, age and sex (higher mean difference in men compared to women).

The DEGS1 data were weighted to the population in Germany as of 31 Dec 2010 with respect to age, sex, region and nationality as well as type of municipality and education. The weighting factor for DEGS1 considered the re-participation probability of the former GNHIES98 participants based on a logistic regression model [17]. The GNHIES98 data were also weighted to the population structure as of 31 Dec 2010, but to the 1998 educational distribution because of the secular changes in education levels. All analyses were weighted, so that the differences observed are controlled for age and sex.

Mean SBP, DBP and the prevalence of hypertensive BP (≥140/90 mmHg) and hypertension (BP ≥140/90 mmHg or taking ATC-coded antihypertensive medication in case of known hypertension) were assessed in both sexes and in subgroups of age, BMI, antihypertensive medical treatment, alcohol consumption, physical activity, smoking status and socioeconomic status (SES) for both surveys. Differences between the surveys were tested with chi-squared tests for categorical variables and t-tests for continuous variables. Tests were considered significant if p ≤ 0.05. To visualize the association of BMI with SBP, scatterplots of BMI and SBP were produced by combining the following two plots in one graph: 1.) BMI was divided into 5 % BMI percentile ranges (<P5, P5-P10 etc. up to P95-P100) and the mean BMI and mean SBP were plotted for these percentile ranges; 2.) curves of predicted SBP values from unadjusted linear regression models of SBP on BMI, including BMI squared (BMI^2^). Generalized linear regression analyses were conducted separately for GNHIES98 and DEGS1 and were stratified by sex (since a BMI x sex interaction was significant, *p* = 0.000) and treatment (all and untreated participants). BMI^2^ was included to allow for a non-linear association of BMI and SBP. The significance of the BMI-SBP-association was tested in a combined test for BMI and BMI^2^.

To investigate whether the BMI-SBP-association changed between 1998 and 2008–11, both surveys were combined and a BMI × survey and BMI^2^ × survey interaction were included in the model. The significance of the interaction was tested in a combined test for BMI × survey and BMI^2^ × survey. Moreover, all analyses were adjusted for selected covariates including age, antihypertensive medication, alcohol consumption, physical activity, smoking status and SES. The analyses were computed with the complex samples option in SPSS 20.0, using the LMATRIX option for the combined tests.

## Results

Selected characteristics of all and untreated study participants 1998 and 2008–2011 are shown in Table 1. Between 1998 and 2008–11, mean SBP decreased by 5 mmHg (from 129.0 mmHg to 124.1 mmHg, *p* = 0.000) in all participants, and in untreated participants by 4 mmHg (from 126.0 mmHg to 122.3 mmHg, *p* = 0.000) (Table 1). In women, mean SBP was lower in 2008–11 by −6.6 mmHg for all women and −5.4 mmHg for untreated women compared to 1998, in men −3.3 mmHg among all men and −2.0 mmHg among untreated men. SBP was positively associated with age at both time points, although the increase in SBP with age was steeper in 1998 than in 2008–11. Moreover, the prevalence of hypertensive BP (≥140/90 mmHg) decreased from 22.8 % in 1998 to 15.3 % in 2008–11 among all and from 15.9 % to 11.4 % among untreated participants (Table 1).

**Table 1:** Selected characteristics of participants aged 18–79 years in 1998 and 2008-11

	All participants													Untreated participants												
	1998						2008-2011							1998						2008-2011						
N	6,931						6,861							5,663						4,755						
	%	95 %-CI					%	95 %-CI					p	%	95 %-CI					%	95 %-CI					p
Men	50.3	(	49.1	-	51.6	)	50.3	(	48.7	-	51.9	)		51.7	(	50.3	-	53.1	)	50.6	(	48.7	-	52.5	)	
Women	49.7	(	48.4	-	50.9	)	49.7	(	48.1	-	51.3	)		48.3	(	46.9	-	49.7	)	49.4	(	47.5	-	51.3	)	
Mean age (years)	47.4	(	46.7	-	48.1	)	47.4	(	46.9	-	47.8	)		43.6	(	43.0	-	44.3	)	42.1	(	41.6	-	42.5	)	
Age groups																										
18-29 years	17.4	(	16.1	-	18.7	)	18.9	(	18.0	-	19.8	)		21.3	(	19.8	-	22.9	)	25.1	(	24.0	-	26.3	)	
30-44 years	26.8	(	25.5	-	28.1	)	25.2	(	24.0	-	26.4	)		31.7	(	30.4	-	33.1	)	31.7	(	30.3	-	33.1	)	
45-64 years	36.6	(	35.3	-	37.9	)	36.4	(	35.0	-	37.8	)		35.6	(	34.2	-	37.1	)	34.7	(	32.9	-	36.5	)	
65-79 years	19.3	(	17.7	-	21.0	)	19.5	(	18.5	-	20.6	)		11.3	(	10.0	-	12.7	)	8.5	(	7.7	-	9.3	)	
BMI class																										
17,5-25 kg/m^2^	38.7	(	37.0	-	40.5	)	40.6	(	39.0	-	42.2	)	0.070	44.1	(	42.1	-	46.1	)	48.6	(	46.6	-	50.6	)	0.000
≥25-30 kg/m^2^	41.0	(	39.6	-	42.3	)	37.3	(	36.0	-	38.7	)	0.000	39.8	(	38.2	-	41.3	)	36.2	(	34.6	-	37.8	)	0.001
≥30-40 kg/m^2^	20.3	(	18.9	-	21.8	)	22.1	(	20.7	-	23.6	)	0.045	16.1	(	14.7	-	17.7	)	15.2	(	13.8	-	16.7	)	0.353
Mean SBP (mmHg)	129.0	(	128.2	-	129.7	)	124.1	(	123.5	-	124.6	)	0.000	126.0	(	125.4	-	126.7	)	122.3	(	121.7	-	122.8	)	0.000
Mean DBP (mmHg)	78.2	(	77.8	-	78.7	)	73.3	(	72.9	-	73.6	)	0.000	77.0	(	76.5	-	77.4	)	72.9	(	72.5	-	73.2	)	0.000
Hypertension prevalence^a^	29.4	(	27.6	-	31.2	)	31.2	(	29.6	-	32.8	)	0.113	/	(	/	-	/	)	/	(	/	-	/	)	/
Prevalence BP ≥140/90 mmHg	22.8	(	21.1	-	24.6	)	15.3	(	14.0	-	16.8	)	0.000	15.9	(	14.5	-	17.4	)	11.4	(	10.1	-	12.8	)	0.000
Antihypertensive medication	19.2	(	17.7	-	20.8	)	25.3	(	24.0	-	26.6	)	0.000	/	(	/	-	/	)	/	(	/	-	/	)	/
Daily smoker	26.5	(	24.9	-	28.1	)	23.7	(	22.2	-	25.2	)	0.001	29.9	(	28.1	-	31.7	)	26.9	(	25.1	-	28.8	)	0.005
Alcohol^b^																										
0 g/d	19.6	(	18.2	-	21.1	)	14.4	(	13.3	-	15.6	)	0.000	17.8	(	16.3	-	19.4	)	14.0	(	12.7	-	15.5	)	0.000
<10/20 g/d	61.2	(	59.6	-	62.9	)	69.5	(	68.0	-	70.9	)	0.000	62.7	(	60.8	-	64.6	)	70.5	(	68.8	-	72.2	)	0.000
>10/20 g/d	19.2	(	17.7	-	20.7	)	16.1	(	15.0	-	17.2	)	0.000	19.5	(	17.9	-	21.2	)	15.5	(	14.1	-	16.9	)	0.000
Sports activity																										
>2 h/week	19.1	(	17.7	-	20.6	)	25.8	(	24.3	-	27.3	)	0.000	21.0	(	19.5	-	22.6	)	27.6	(	25.9	-	29.4	)	0.000
<2 h/week	32.0	(	30.6	-	33.5	)	41.6	(	40.0	-	43.1	)	0.000	33.9	(	32.3	-	35.5	)	42.0	(	40.2	-	43.9	)	0.000
no sports activity	48.9	(	46.9	-	50.9	)	32.7	(	31.1	-	34.3	)	0.000	45.1	(	43.2	-	47.1	)	30.4	(	28.6	-	32.2	)	0.000
Socioeconomic status (SES)																										
low	19.4	(	17.6	-	21.3	)	19.6	(	18.0	-	21.2	)	0.834	18.3	(	16.5	-	20.3	)	18.7	(	17.1	-	20.4	)	0.726
medium	60.5	(	59.0	-	62.1	)	60.4	(	58.7	-	62.1	)	0.905	60.9	(	59.1	-	62.6	)	59.7	(	57.7	-	61.7	)	0.320
high	20.1	(	18.2	-	22.2	)	20.0	(	18.4	-	21.8	)	0.929	20.8	(	18.9	-	22.9	)	21.6	(	19.7	-	23.6	)	0.445

Hypertension prevalence was defined as BP ≥140/90 mmHg or antihypertensive medication use in case of physician-diagnosed known hypertension

Alcohol intake in gram/day (g/d) was categorized on basis of the upper tolerable intake level (UL). The alcohol UL is set on 10 g/d for women and 20 g/d for men

During the same period, mean BMI remained constant at a high level (around 27 kg/m^2^ in men and 26 kg/m^2^ in women). The prevalence of obesity slightly increased in men from 19.0 % to 22.4 % (*p* = 0.006) and remained at around 22 % (*p* = 0.912) in women. In both surveys, the obesity prevalence increased continuously with age in women, but it reached a plateau at the age of 60–69 years in men (data not shown). Obesity prevalence in the untreated group did not change over time (16.1 % in 1998 and 15.2 % in 2008–11, *p* = 0.353) and was lower at both times than in the overall group (Table 1).

The decrease in mean SBP over time was more pronounced in overweight (−4.9 mmHg) and obese persons (−6.9 mmHg) than in non-overweight participants (−3.7 mmHg). Untreated overweight (−3.5 mmHg) and obese (−4.3 mmHg) participants also showed a greater decrease in mean SBP than non-overweight ones (−3.1 mmHg). The prevalence of hypertensive BP (≥140/90 mmHg) decreased by more than one third among all BMI groups (Table 2). Nevertheless, overweight and obese individuals still had a notably higher mean SBP and higher prevalence of hypertensive BP in 2008–11 than non-overweight individuals (Table 2).

**Table 2:** Blood pressure by BMI class in all and untreated participants in 1998 and 2008-11

	SBP						DBP						Prevalence BP ≥140/90 mmHg	Treated
1998														
	mmHg	95 %-CI					mmHg	95 %-CI					%	%
All participants														
17.5-25 kg/m^2^	123.4	(	122.4	-	124.3	)	75.2	(	74.6	-	75.7	)	12.3	8.0
≥25-30 kg/m^2^	131.1	(	130.2	-	132.0	)	79.2	(	78.6	-	79.7	)	26.2	21.6
≥30-40 kg/m^2^	135.4	(	134.2	-	136.7	)	82.2	(	81.6	-	82.9	)	35.9	35.8
Untreated participants														
17.5-25 kg/m^2^	121.8	(	121.0	-	122.6	)	74.5	(	73.9	-	75.0	)	8.6	/
≥25-30 kg/m^2^	128.3	(	127.5	-	129.2	)	78.1	(	77.6	-	78.7	)	19.2	/
≥30-40 kg/m^2^	131.9	(	130.7	-	133.1	)	80.9	(	80.2	-	81.6	)	27.5	/
2008-11														
All participants														
17.5-25 kg/m^2^	119.7	(	119.1	-	120.3	)	70.8	(	70.3	-	71.2	)	8.0^a^	10.5^a^
≥25-30 kg/m^2^	126.2	(	125.5	-	127.0	)	74.7	(	74.2	-	75.2	)	17.9^a^	27.6^a^
≥30-40 kg/m^2^	128.5	(	127.3	-	129.6	)	75.4	(	74.7	-	76.1	)	24.4^a^	48.5^a^
Untreated participants														
17.5-25 kg/m^2^	118.7	(	118.0	-	119.4	)	70.4	(	69.9	-	70.8	)	6.5^a^	/
≥25-30 kg/m^2^	124.8	(	124.0	-	125.7	)	74.8	(	74.2	-	75.4	)	14.3^a^	/
≥30-40 kg/m^2^	127.6	(	126.3	-	129.0	)	76.3	(	75.4	-	77.3	)	20.2^a^	/

^a^p significant <0.05 for survey difference 1998 vs. 2008–11

The proportion of persons taking antihypertensive medication (irrespective of indication) augmented significantly from 19.2 % in 1998 to 25.3 % in 2008–11 (*p* = 0.000; Table 1). Thereby, the treatment proportion was higher in overweight and obese persons than in non-overweight individuals (Table 2), whereby 35.8 % of obese persons were treated in 1998 and almost half of all obese (48.5 %) received antihypertensives in 2008–11 (*p* = 0.000). However, the relative increase in treatment over time amounted to approximately 30 %, irrespective of BMI group.

**Fig. 1:**
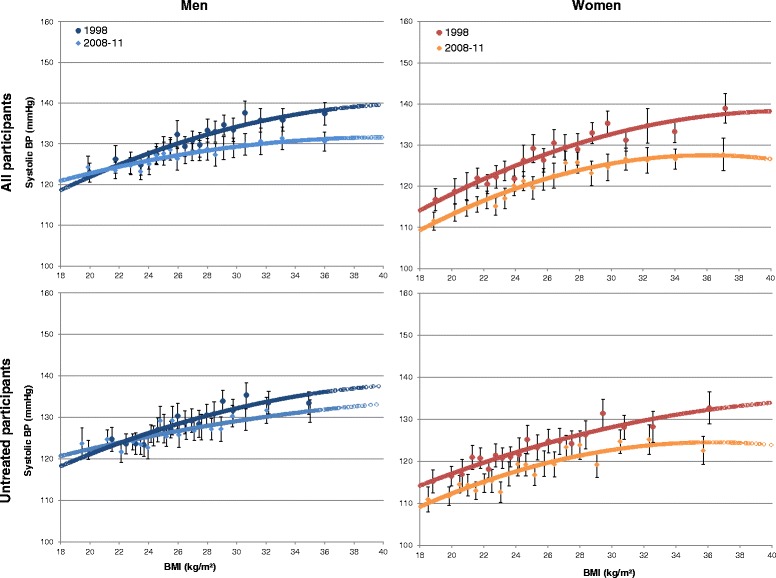
Mean systolic blood pressure for participants within BMI ranges defined by BMI percentiles. BMI percentile ranges: *P* < 5, P5-P10, P10-P15 up to P95-P100. Curves represent predicted SBP values from univariable generalized linear regression models

Figure 1 illustrates the association of BMI with SBP separately for all and untreated men and women in 1998 and 2008–11. Mean SBP was plotted against BMI for subgroups defined by narrow BMI percentile ranges. Following from these plots, BMI was positively associated with SBP in all and untreated participants at both times, yet the association tended to level out with increasing BMI suggesting a non-linear association of BMI and SBP. Table 3 shows the unadjusted linear regression models of SBP and BMI, which included BMI^2^. BMI^2^ thereby was significant at the *p* < 0.05 level for all men and all women in 1998 as well as all and untreated women in 2008–11 (not significant for all and untreated men in 2008–11 as well as untreated men and women in 1998, data not shown). The regression curves from these models are also shown in Fig. 1. The level of SBP for a given BMI was generally lower in 2008–11 than in 1998. The association between BMI and SBP was also compared across age groups (18–39 years, 40–59 years, 60–79 years) in an age-stratified analysis (Additional file 1) as well as by adding an interaction term of BMI × agegroup and BMI^2^ × agegroup to all models described above. These interactions were not statistically significant (combined p > 0.05) in any model but one, i.e. “all men” in 2008–11. The association of BMI and SBP by age group in untreated men and women is graphically depicted in an additional figure (Additional file 2).

**Table 3:** Association between BMI and systolic blood pressure in 1998 and 2008-11

	1998															
	Men								Women							
	ß		95 %-CI				combined p	R^2^	ß		95 %-CI				combined p	R^2^
All participants																
Unadjusted								0.066								0.112
BMI	2.939	(	1.196	-	4.683	)	0.000		3.700	(	2.293	-	5.107	)	0.000	
BMI^2^	−0.034	(	−0.066	-	−0.003	)			−0.045	(	−0.070	-	−0.020	)		
Adjusted^a^								0.216								0.374
BMI	1.013	(	−0.716	-	2.742	)	0.000		0.351	(	−0.874	-	1.576	)	0.000	
BMI^2^	−0.007	(	−0.038	-	0.024	)			0.002	(	−0.021	-	0.024	)		
Untreated participants																
Unadjusted								0.064								0.086
BMI	2.512	(	0.789	-	4.235	)	0.000		2.419	(	0.960		3.878	)	0.000	
BMI^2^	−0.028	(	−0.060	-	0.003	)			−0.026	(	−0.053		0.001	)		
Adjusted								0.195								0.299
BMI	0.571	(	−1.088	-	2.229	)	0.000		−0.481	(	−1.935		0.972	)	0.000	
BMI^2^	0.003	(	−0.027	-	0.033	)			0.019	(	−0.008		0.046	)		
	2008-11															
All participants																
Unadjusted								0.027								0.098
BMI	1.722	(	0.443	-	3.000	)	0.000		4.048	(	2.613	-	5.483	)	0.000	
BMI^2^	−0.021	(	−0.044	-	0.001	)			−0.056	(	−0.083	-	−0.030	)		
Adjusted								0.056								0.221
BMI	0.728	(	−0.641	-	2.097	)	0.000		1.943	(	0.727	-	3.159	)	0.000	
BMI^2^	−0.006	(	−0.029	-	0.018	)			−0.026	(	−0.048	-	−0.003	)		
Untreated participants																
Unadjusted								0.037								0.085
BMI	1.326	(	−0.175	-	2.827	)	0.000		3.327	(	1.719	-	4.936	)	0.000	
BMI^2^	−0.013	(	−0.040	-	0.014	)			−0.046	(	−0.077	-	−0.015	)		
Adjusted								0.088								0.203
BMI	0.699	(	−0.864	-	2.261	)	0.000		1.433	(	0.032	-	2.834	)	0.000	
BMI^2^	−0.003	(	−0.030	-	0.025	)			−0.014	(	−0.040	-	0.013	)		
	1998 and 2008–11 combined															
All participants																
Unadjusted								0.061								0.140
BMI	2.939	(	1.196	-	4.683	)	0.000		3.700	(	2.293	-	5.107	)	0.000	
BMI^2^	−0.034	(	−0.066	-	−0.003	)			−0.045	(	−0.070	-	−0.020	)		
BMI x survey^b^	−1.218	(	−3.251	-	0.815	)	0.000		0.348	(	−1.589	-	2.284	)	0.019	
BMI^2^ x survey	0.013	(	−0.024	-	0.049	)			−0.011	(	−0.046	-	0.024	)		
Adjusted								0.132								0.319
BMI	1.800	(	0.121	-	3.479	)	0.000		1.003	(	−0.220	-	2.226	)	0.000	
BMI^2^	−0.018	(	−0.048	-	0.012	)			−0.007	(	−0.029	-	0.016	)		
BMI x survey	−1.752	(	−3.637	-	0.132	)	0.000		0.343	(	−1.252	-	1.938	)	0.006	
BMI^2^ x survey	0.022	(	−0.012	-	0.056	)			−0.011	(	−0.040	-	0.018	)		
Untreated participants																
Unadjusted								0.058								0.116
BMI	2.512	(	0.789	-	4.235	)	0.000		2.419	(	0.960	-	3.878	)	0.000	
BMI^2^	−0.028	(	−0.060	-	0.003	)			−0.026	(	−0.053	-	0.001	)		
BMI x survey	−1.186	(	−3.356	-	0.984	)	0.005		0.908	(	−1.136	-	2.953	)	0.330	
BMI^2^ x survey	0.015	(	−0.024	-	0.055	)			−0.020	(	−0.058	-	0.019	)		
Adjusted								0.141								0.274
BMI	1.260	(	−0.399	-	2.919	)	0.000		0.007	(	−1.399	-	1.413	)	0.000	
BMI^2^	−0.008	(	−0.038	-	0.022	)			0.011	(	−0.015	-	0.037	)		
BMI x survey	−1.089	(	−3.045	-	0.867	)	0.011		0.921	(	−0.994	-	2.837	)	0.560	
BMI^2^ x survey	0.014	(	−0.021	-	0.050	)			−0.016	(	−0.052	-	0.020	)		

^a^ Adjusted for: age in 5 year intervals, antihypertensive medication, alcohol intake, sports activity, smoking status, socioeconomic status

^b^ The combined models include survey year. Survey was coded as 0 = 1998, 1 = 2008–11

The strength of the BMI-SBP-association decreased over time in men (both all and untreated) as shown in a regression analysis with combined data from the two surveys which included an interaction term of BMI × survey (Table 3). This weakening was also observed after adjustment for age, antihypertensive medication, alcohol intake, sports activity, smoking status and SES. In women, the association became weaker only in the overall analysis including treated participants (both unadjusted and adjusted). No change of the BMI-SBP- association was observed in untreated women (unadjusted and adjusted) (Tables 3 and 4).

In a further analysis, the association of BMI and SBP was assessed only in untreated participants with hypertension. In this group, however, BMI was no longer associated with SBP neither in 1998 nor in 2008–11 (Additional file 3).

Finally, Table 4 exemplifies the SBP increase with rising BMI separately for men and women, estimated from the unadjusted regression models. Accordingly, in 1998 a BMI increase from 20 kg/m^2^ to 25 kg/m^2^ was associated with an estimated SBP higher by 7.0 mmHg in men and 8.4 mmHg in women, whereas a BMI increase from 30 kg/m^2^ to 35 kg/m^2^ was linked to a 3.6 mmHg and 3.9 mmHg higher SBP in men and women, respectively. In 2008–11, SBP increased less with BMI, especially in men, where SBP was higher by 3.8 mmHg for a BMI augmenting from 20 kg/m^2^ to 25 kg/m^2^ and 1.7 mmHg for 30 kg/m^2^ to 35 kg/m^2^. The corresponding estimates for women were 7.6 mmHg and 2.0 mmHg. A similar pattern was seen in untreated participants, although the estimated increase in SBP was less than among all participants (e.g. men in 1998, BMI increase 20 to 25 kg/m^2^: 6.2 mmHg, 2008–11: 3.7 mmHg; Table 4)

**Table 4:** Estimated increase in systolic blood pressure associated with an increase in BMI in 1998 and 2008-11

	1998		2008	
	Estimated SBP increase			
	All participants	Untreated participants	All participants	Untreated participants
BMI increase				
Men				
20 to 25 kg/m^2^	7.0	6.2	3.8	3.7
25 to 30 kg/m^2^	5.3	4.8	2.7	3.0
30 to 35 kg/m^2^	3.6	3.4	1.7	2.4
Women				
20 to 25 kg/m^2^	8.4	6.2	7.6	6.3
25 to 30 kg/m^2^	6.2	4.9	4.8	4.0
30 to 35 kg/m^2^	3.9	3.6	2.0	1.7

SBP increase was estimated based on unadjusted generalized linear regression models

## Discussion

This study shows that in Germany both in 1998 and in 2008–11 SBP is still associated with BMI both in the overall general adult population and in the population not taking antihypertensive medication. Thus, we cannot confirm previous findings about a disappearance of the BMI-SBP-association at the population level in worldwide data [13]. We did however find a weakening of the association over time in the overall samples of men and women as well as in untreated men, while in untreated women the association did not change significantly.

The Global Burden of Metabolic Risk Factors of Chronic Diseases Project analyses had shown a decoupling of the BMI-SBP-association between 1980 and 2008 on a global scale as well as for high-income regions like Western Europe and North America based on country-level aggregated data from 199 nations. A one unit higher mean population BMI was associated with a 1.35 mmHg (CI 1.05 - 1.80) higher mean SBP in women and 1.19 mmHg (CI 1.01 - 1.54) in men in 1980, but this positive association seemed to have disappeared in 2008 [13]. However, the analyses did not account for other influential factors like antihypertensive medication use and were constrained to a linear analysis of population means.

The findings of our study are in line with analyses of two cross-sectional surveys from the Seychelles comparing the BMI-SBP association in 1989 and 2004. In these studies a significant decline in the strength of the adjusted association of BMI and BP was observed and was not explained by the increased proportion of persons treated with antihypertensives. Among untreated participants, a one-unit BMI increase was thereby associated with a 1.98 mmHg higher SBP in 1989 but only 1.27 mmHg in 2004 after multivariable adjustment. Furthermore, a significant weakening but not decoupling of the BMI-SBP-association was also found for 9- to 11-year-old U.K. children when comparing 1980 and 2008 data [15]. In contrast, other recent studies did not support a decreasing association of BMI and SBP in adults in France [22], Taiwan [23], Switzerland [24] as well as in both children and adults in the Seychelles [24, 25].

In this study, we found no association of BMI and SBP within the group of untreated participants with hypertension at both times. This finding is consistent with a study which investigated the relationship of adiposity to BP in African-American adults from Milwaukee as well as non-Hispanic black and white adults from the 1999 to 2004 National Nutrition and Health Examination Survey (NHANES) waves. Here, BMI was significantly associated with BP in normotensives but not in untreated hypertensives among both Milwaukee and NHANES participants. Even more, BMI was significantly associated with SBP only in the lowest quartile of BP (SBP ≤ 115, DBP ≤ 89) if normotensive and untreated hypertensive persons were assessed together, which means, conversely, that already in the upper ranges of the normotensive BP distribution, BMI was no longer associated with SBP [26].

Since overweight and obese persons are more likely treated with antihypertensives than normalweight persons in our study, other influential factors (e.g. genetic and environmental factors) than weight may have contributed to BP elevation in this group of untreated hypertensives.

In Germany, trends of SBP and BMI over time differed. While mean SBP and the prevalence of high BP ≥140/90 mmHg decreased substantially in Germany between 1998 and 2008–11 both in the overall sample and in untreated persons, mean BMI remained constant on a high level and the prevalence of obesity even slightly increased in men. These developments were accompanied by an improved management of hypertension and a considerable increase in antihypertensive medication use. Worldwide, the Global Burden of Metabolic Risk Factors of Chronic Diseases project estimated a decrease in mean SBP of −0.8 mmHg per decade in men and −1.0 mmHg in women for the period from 1980 to 2008. In high-income regions, like Western Europe and North America, the estimated decrease was even higher and amounted to −2.1 mmHg and −3.5 mmHg in Western European men and women, respectively, and −2.8 mmHg in men and −2.3 mmHg in women from North America [13]. In the same analysis, estimated mean BMI increased globally by 0.4 kg/m^2^ per decade in men and 0.5 kg/m^2^ in women. In 2008, U.S. men and women thereby had the highest mean BMI among high-income countries and U.S. women also displayed the greatest BMI gain of 1.2 kg/m^2^ per decade. In contrast, Western Europe displayed a modest BMI increase of 0.6 kg/m^2^ per decade in men and 0.4 kg/m^2^ in women.

Among children, a decrease in SBP but increase in BMI and in the prevalence of overweight and obesity were observed in some studies [25, 27, 28], whereas both SBP and BMI increased in other paediatric populations [15, 29–31]. Diverging trends in the development of SBP and BMI were observed in many populations [10, 14, 32, 33], although an increase of both SBP and BMI was also observed, in particular for some developing countries [30, 31].

In summary, in congruence with other high-income nations, mean SBP decreased in Germany during the last decade, whereas mean BMI remained constant and obesity prevalence in men increased. Concomitantly, the association of BMI and SBP became weaker. In part, the weakening of the BMI-SBP-association may be attributable to improvements in diagnosis and treatment of high BP.

Firstly, overweight and obese persons were more often treated with antihypertensives in 2008–11 than in 1998. More than one fourth of all overweight and half of all obese were treated in 2008–11 and thus, the distribution of SBP was truncated in a significant proportion of the overweight population. In addition, since the treatment prevalence was significantly higher in 2008–11 than in 1998, this could have contributed to a decreased association. Secondly, the hypertension threshold had been lowered to 140/90 mmHg only shortly before the 1998 survey [34]. It is likely that the new guidelines were better implemented in 2008–11 than in 1998, i.e. more patients were treated at lower BP levels in 2008–11, leaving a healthier group untreated in 2008–11 and thus contributing to a decreased BMI-SBP-association also among untreated persons.

It was argued by some authors that the correlation of SBP and BMI observed in previous decades may have been overestimated due to inappropriate BP cuffs in the obese (undercuffing leading to overestimation of BP) [24, 35, 36]. The cuffs differed between GNHIES98 and DEGS1, although both are in line with current BP measurement guidelines [37]. As reported previously [21], this was investigated in a separate study with sequential measurements with the GNHIES98 and DEGS1 devices and cuffs. A calibration formula was derived which accounted not only for device differences but also for the different ratios of cuff width and arm circumference [21]. It is therefore not likely that changes in the SBP-BMI- association between the two surveys are due to cuff differences in the obese.

The strengths of this analysis include the nationwide population-based samples from two national health surveys with largely identical sampling and measurement methods, the standardisation of BP, weight and height measurements and the availability of high-quality medication data. It is a limitation of our analysis that we could not adjust for the intake of salt, fruit and vegetables which are related to both BMI and BP. These parameters were either not measured or lacked comparability between the two surveys. We included other possibly influential lifestyle factors like sports activity, alcohol consumption and smoking. However, their prevalence did not change considerably between 1998 and 2008–11 and adjustment for these factors did not change the BMI-SBP-association very much compared to adjustment for age only (data not shown).

## Conclusions

For Germany, our analysis suggests a decrease in the cross-sectional association of BMI and SBP particularly in men which is in part explained by improvements in the diagnosis and treatment of high BP. However, the association has not disappeared as suggested by some analyses from other countries. BMI remains significantly associated with BP at the population level over a broad BMI range. This suggests that a population-wide shift of the BMI distribution towards lower BMI values should remain a prior public health issue.

## Additional files

Additional file 1.
**Unadjusted association between BMI and systolic blood pressure in 1998 and 2008–11 stratified by age.** The combined models include survey year. Survey was coded as 0 = 1998, 1 = 2008–11. The table displays the unadjusted association of BMI with SBP for the age groups 18–39 years, 40–59 years and 60–79 years separately for 1998 and 2008–11 and in a combined dataset of both surveys. Additional file 2.
**Association of BMI with systolic blood pressure in different age groups from age-group specific linear regression models including BMI and BMI squared.** The range between the 5th and 95th BMI percentile for each age group is plotted. The p-value is the combined p for the interaction terms of age-group with BMI and with BMI squared from the respective model including all age-groups. The figure illustrates the predicted SBP derived from unadjusted linear regression models including BMI and BMI^2^ stratified by age groups 18–39, 40–59 and 60–79 years plotted against narrow BMI steps ranging from the 5th to the 95th BMI percentile for each age group. (PDF 34 kb) Additional file 3.
**Unadjusted association between BMI and systolic blood pressure in untreated hypertensives in 1998 and 2008–11.** The combined models include survey year. Survey was coded as 0 = 1998, 1 = 2008–11. The table displays the unadjusted association of BMI with SBP in untreated persons with hypertension separately for 1998 and 2008–11 and in a combined dataset of both surveys.

## Abbreviations

ATC: Anatomic Therapeutic Chemical Classification System
BMI: Body mass index
BP: Blood pressure
DBP: Diastolic blood pressure
DEGS1: German Health Interview and Examination Survey for Adults 2008–2011
GNHIES98: German National Health Interview and Examination Survey 1998
SBP: Systolic blood pressure
