# Supplementary figures and images for: Change in the association of body mass index and systolic blood pressure in Germany – national cross-sectional surveys 1998 and 2008–2011
Source: BMC Public Health. 2015 Jul 25;15:705. doi: 10.1186/s12889-015-2023-8 (PMC4514940; doi:10.1186/s12889-015-2023-8)

## Men

1998

$p = 0.072$

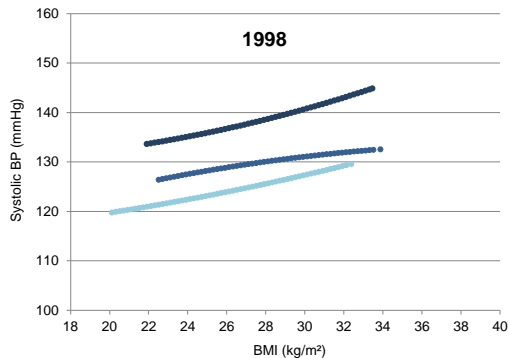

## Women

1998

$p = 0.470$

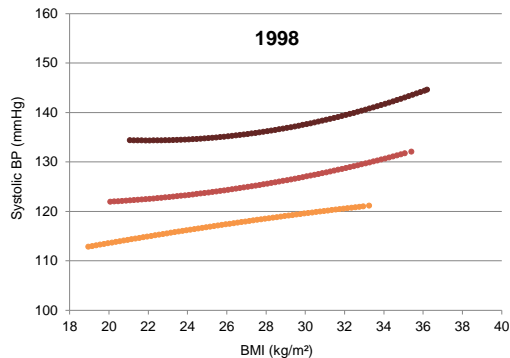

2008-11

$p = 0.304$

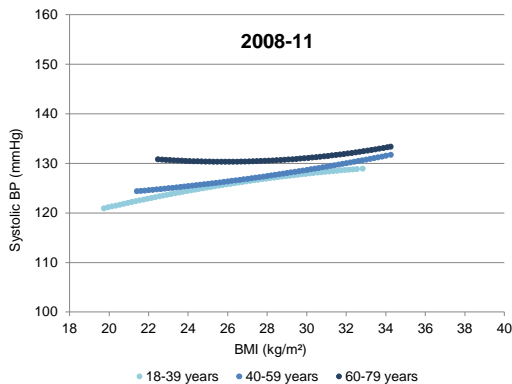

2008-11

$p = 0.098$

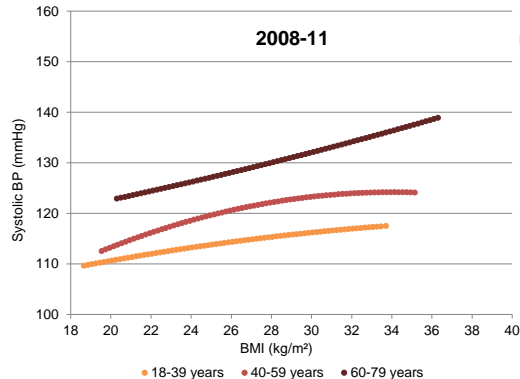

Supplement: Additional file 2. — Association of BMI with systolic blood pressure in different age groups from age-group specific linear regression models including BMI and BMI squared. The range between the 5th and 95th BMI percentile for each age group is plotted. The p-value is the combined p for the interaction terms of age-group with BMI and with BMI squared from the respective model including all age-groups. The figure illustrates the predicted SBP derived from unadjusted linear regression models including BMI and BMI2 stratified by age groups 18–39, 40–59 and 60–79 years plotted against narrow BMI steps ranging from the 5th to the 95th BMI percentile for each age group. (PDF 34 kb) [file 12889_2015_2023_MOESM2_ESM.pdf]
